# Report of the Case of the Late Com. Jesse D. Elliott

**Published:** 1846-05

**Authors:** E. K. Kane

**Affiliations:** Assistant Surgeon, U. S. Navy


### MEDICAL EXAMINER,

AND

### RECORD OF MEDICAL SCIENCE.

NEW SERIES.—No. XVII.—MAY, 1846.

### ORIGINAL COMMUNICATIONS.

*Report of the Case of the late Com. Jesse D. Elliott.* By E. K. Kane, M. D., Assistant Surgeon, U. S. Navy. (Communicated by Dr. Kearney.)

*Philadelphia, Feb. 25th,* 1846.

*Sir*—I submit the subjoined abstract of a post mortem examination of the body of Commodore Jesse Duncan Elliott, made by your direction, on the 11th of December last, in the presence of the several Surgeons of the Navy, then in Philadelphia.

The examination was confined to the viscera and appendages of the thorax and abdomen, and took place thirty-two hours after death. For more than two weeks previous to this event, the deceased had preserved a nearly upright posture in an arm chair. His age was 62 years and four months.

General aspect somewhat cachetic, emaciation excessive : parietes of chest prominent ; integuments of abdomen voluminous and flabby ; superior extremities flexible ; inferior, œdematous and distended.

Thorax.—No anterior adhesions. *Right Lung,* in natural position ; active pneumonic engorgement passing into red hepatization, throughout its upper third ; middle portions free, and crepitant ; outer margin of inferior lobe, bladed and solidified by compression. *Left Lung* : extensive but old pleuritic adhesions along inferior face of superior lobe ; plastic bands of recent lymph between the pendulous portion above the interlobular fissure and the pericardium contiguous; inferior lobe compressed, atrophied, and nearly concealed by the distended pericardium. Free serum in cavity of chest, about Oiij. *Pericardium* dilated and protruding, recent adhesions between its upper face and the left lung, as before mentioned. Effused fluid ℥xi. *Heart:* general hypertrophy with dilatation of the cavities; left ventricle disproportionately involved both as to walls and cavity; substance of viscus pale and flabby; circumference around auriculo-ventricular groove, thirteen inches; apex forced obliquely upwards to the junction of fifth rib with sternum; *aortic valves*, the seat of cartilaginous vegetations, with slight ossific deposites above the corpuscles; general insufficiency with marked attenuation of their marginal curves; contiguous aorta large, but not perceptibly modified in its tunics; mitral and pulmonary valves healthy and free; chordæ tendinæ unusually developed, but not the "shortening and thickening" of authors. Weight ℥xiv.

Abdomen.—Integuments thickly lined with fat; tissues much modified by serous infiltration. *Liver*, diminished in bulk, with general condensation of structure; anterior face of right lobe slightly depressed; peritoneal envelope unaltered. Parenchyma evincing the adipose transformation, unctuous to touch, greasing the scalpel, and imparting oily stain to bibulous paper. Colour tawney yellow with brownish patches about the fissures; cirrhosed protrusions on cut surfaces, with general evidences of granular degeneration. *Stomach,* distended and somewhat exceeding the natural size; mucous lining in the neighbourhood of larger curvature attenuated, and softened; marked hypertrophy and induration of mucous and sub-mucous cellular tissues about lesser curve, which increased towards pyloric orifice with alternating patches of slaty softening and diffuse redness. *Note.*—The hypertrophy of the mucous and subjacent tissues exhibited some traces of scirrhoid degeneration, and was sufficiently extensive to interfere with the passage of chyme into the duodenum. *Duodenum*, much stained by biliary secretion; mucous coat of large and small intestines *darker* in colour than usual; mesenteric glands unchanged. *Spleen,* somewhat condensed in structure, and about the medium size; weight ℥vii. *Kidneys,* natural as to consistence and colour, the distinction between cortical and tubular portions being remarkably well defined.

The case in its progress presented few variations from the ordinary routine of visceral dropsies, the active disease of the lung and pleura being regarded as merely induced complications.

The structural alterations of most importance were those of the liver and heart, the atrophy of the former, and the valvular insufficiency of the latter organ, having an interesting connexion, not only with the phenomena of effusion, but with sundry unexplained symptoms connected with the circulation and respiration. In order of sequence, the liver was probably the first organ involved, and, in confirmation of the history of the case, must have dated some ten years antecedent to its termination; the affection of the stomach assuming a permanent form at a more recent period.

It may be well to lay some stress upon the fact, that the compensating action of the kidneys continued, to the last, unimpaired.

Very respectfully,
Your obd't. ser't,
E. K. Kane,
Assistant Surgeon, U. S. N.

John A. Kearney, M. D., U. S. N.,
President of Examining Board.

